# Light chain (AL) amyloidosis: update on diagnosis and management

**DOI:** 10.1186/1756-8722-4-47

**Published:** 2011-11-18

**Authors:** Michael Rosenzweig, Heather Landau

**Affiliations:** 1 City of Hope National Cancer Center, Duarte, California, USA; 2 Memorial Sloan- Kettering Cancer Center Department, New York, New York, USA

## Abstract

Light chain (AL) amyloidosis is a plasma cell dyscrasia characterized by the pathologic production of fibrillar proteins comprised of monoclonal light chains which deposit in tissues and cause organ dysfunction. The diagnosis can be challenging, requiring a biopsy and often specialized testing to confirm the subtype of systemic disease. The goal of treatment is eradication of the monoclonal plasma cell population and suppression of the pathologic light chains which can result in organ improvement and extend patient survival. Standard treatment approaches include high dose melphalan (HDM) followed by autologous hematopoietic stem cell transplantation (SCT) or oral melphalan with dexamethasone (MDex). The use of novel agents (thalidomide, lenalidomide and bortezomib) alone and in combination with steroids and alkylating agents has shown efficacy and continues to be explored. A risk adapted approach to SCT followed by novel agents as consolidation reduces treatment related mortality with promising outcomes. Immunotherapeutic approaches targeting pathologic plasma cells and amyloid precursor proteins or fibrils are being developed. Referral of patients to specialized centers focusing on AL amyloidosis and conducting clinical trials is essential to improving patient outcomes.

## Introduction

Primary systemic or light chain amyloidosis (AL) is characterized by a clonal population of plasma cells in the bone marrow that produce monoclonal light chain of kappa or lambda type. Amyloidogenic light chains misfold forming a highly ordered beta pleated sheet configuration which is the structure that defines amyloid fibrils of any type (including light chain, hereditary, senile systemic or secondary). Contiguous beta pleated sheets wind together into a fibrillar configuration instead of the typical alpha helical pattern of most proteins [1]. Amyloid fibrils deposit in organs, progressively interfering with organ structure and function [2-4]. Commonly affected organs include the heart, kidneys, gastrointestinal (GI) tract/liver or the peripheral or autonomic nervous system (NS).

AL amyloidosis should be suspected in any patient with a monoclonal gammopathy and unexplained shortness of breath, fatigue, edema, weight loss, orthostasis or paresthesias (Table 1)[5]. However, it often requires an astute clinician because symptoms are diverse and easily mimicked by more common disorders. Once considered, the evaluation for AL amyloidosis includes testing to identify an underlying clonal plasma cell disorder (bone marrow aspirate/biopsy, serum and urine electrophoreses and immunofixation and serum free light chain testing). In addition, it is essential to determine organs of involvement and an initial work up should include echocardiogram, EKG, 24 hour urine total protein assessment, orthostatic blood pressures; specific GI and NS testing should be performed if indicated. Confirmation of amyloidosis requires tissue sampling to demonstrate congophilic amyloid deposits or fibrils that are 7-10 nanometers in diameter by electron microscopy. While in some patients amyloid deposition will be identified on bone marrow biopsy, in combination with fat pad aspirate, amyloid deposition can be demonstrated in 85% of patients [6]. However, because there remains a 15% chance that amyloidosis is present even when both the bone marrow and fat pad are negative, involved organs should be biopsied if the index of suspicion is high.

**Table 1:** Reasons to Suspect AL Amyloidosis

1.	Non-diabetic nephrotic syndrome
2.	Non-ischemic cardiomyopathy and hypertrophy*
3.	Hepatomegaly or increased alkaline phosphatase**
4.	Monoclonal gammopathy with
	a. Autonomic or sensory neuropathy
	b. Unexplained fatigue
	c. Edema
	d. Unintentional weight loss

Signs and symptoms that may represent AL amyloidosis in a patient. Non-diabetic nephrotic syndrome; non-ischemic cardiomyopathy and hypertrophy on echocardiogram, especially in the absence of hypertension*; hepatomegaly or increased alkaline phosphatase with no liver abnormalities by imaging**; or autonomic neuropathy with orthostasis or sensory neuropathy with a monoclonal protein. In patients with monoclonal gammopathy and unexplained fatigue, edema, weight loss or paresthesias, AL amyloidosis should also be considered.

Although AL amyloidosis is the most common form of systemic amyloidosis, up to 10% of patients may present with "secondary" or "hereditary" amyloidosis and an incidental monoclonal gammopathy of undetermined significance (MGUS) rather than AL amyloidosis [7]. All amyloid fibrils regardless of their protein of origin intercalate Congo red stain, demonstrate apple-green birefringence under light microscopy and have similar ultrastructural characteristics by electron microscopy. In any patient with more than one source of amyloid, it is essential to determine with certainty the protein composition of the amyloid deposit which may be amyloid A or transthyretin in secondary or hereditary amyloid, respectively. Immunohistochemistry, while routinely used to type amyloid deposits is often unreliable [8,9]. Immunogold electron microscopy is more specific than immunohistochemistry and can be performed on a fat pad samples if amyloid is present and the appropriate antibodies are available [10,11]. However, using laser microdissection with mass spectrometry, all known types of amyloid can be identified with a single test and this method is most reliable [12]. However, this technology is only available at specialized centers. Treatment for AL systemic amyloidosis which is distinctly different from therapy for hereditary variants or secondary amyloidosis, should only be considered once the precursor protein is identified with certainty.

**Figure 1:**
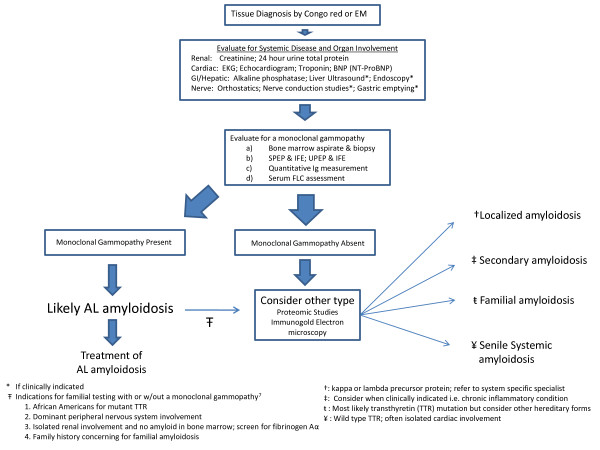
**Evaluation following tissue diagnosis of amyloid**.

In the absence of clonal plasma cells in the bone marrow, light chain amyloid may localized to a single site, most often the skin, larynx or urinary tract [13]. Isolated pulmonary nodules and colonic polyps may represent localized rather than systemic disease [14]. Localized amyloidosis does not require systemic therapy and symptoms should be managed by system specific specialists. An algorithm for the work up of a patient once amyloid is identified by biopsy is shown (Figure 1).

The source of the amyloidogenic light chains is a clone of plasma cells, histologically identical to those seen in the more common plasma cell dyscrasia, multiple myeloma. Given these similarities, to date, treatments for AL have been largely derived from those studied for the treatment of multiple myeloma. However, amyloid- specific research is urgently needed because patients with AL amyloidosis often do not tolerate therapy at doses used for patients with multiple myeloma due to amyloid-affected organ dysfunction. At the present time, there are no drugs specifically FDA-approved for the treatment of amyloidosis.

## Goals of Therapy and Prognostic Markers

**Figure 2:**
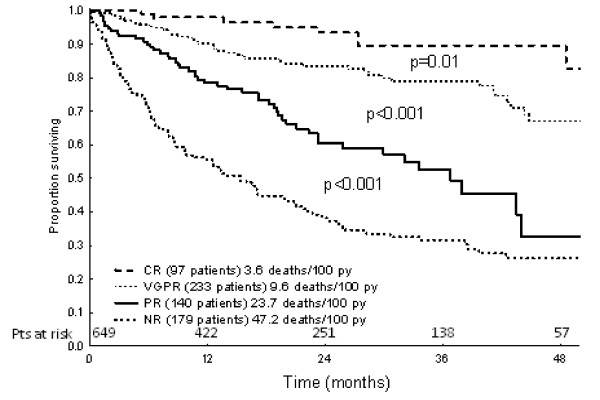
**Survival in patients with AL amyloidosis by hematologic response (adapted with permission from Dr. Pallidini) **[20].

Current therapies that are available for AL amyloidosis are aimed at eradicating the pathologic plasma cells and eliminating the circulating free light chain. Interruption of precursor protein production can lead to the regression of amyloid deposits, organ improvement and extended survival [15-17]. The efficacy of a treatment can be measured both in terms of reduction in the burden of clonal plasma cell disease (hematologic response) and by improvement in the organ function (organ response)[18]. The serum free light chain (FLC) assay which detects circulating FLCs rather than intact immunoglobulins, is a more powerful predictor of survival in AL amyloidosis than standard immunoelectrophoresis [19]. In 2010, the International Society for Amyloidosis revised and validated hematologic response criterion for AL amyloidosis based on FLC assessment at baseline and following treatment (Figure 2)[20]. While complete response (CR) continues to require a negative serum and urine immunofixation electrophoresis (IFE), normal serum FLC ratio and < 5% clonal plasma cells on bone marrow studies, the definitions of partial response (PR) and very good partial response (VGPR) are based on the difference between involved and uninvolved FLC (dFLC). Because ≥ VGPR, defined as dFLC < 40 mg/L is associated with an 80% OS at 3 years, clinicians should adapt therapy in patients who fail to achieve this goal.

Cardiac biomarkers were studied in AL amyloidosis because the extent of cardiac involvement may be the most important determinant of outcome. Troponin I or T provide a quantitative assessment of cardiac damage and BNP and/or NT-proBNP indicate cardiomyocyte stress and are independently associated with survival [21]. By using these biomarkers, a staging system has been developed has been developed and patients can be classified as having stage I, II or III disease with survivals of 26, 11 and 3.5 months, respectively [22]. This staging system is important for clinical management, but also for stratifying patients enrolled on clinical trials.

## Therapeutic options in AL Amyloidosis

### High-dose melphalan and autologous stem cell transplant

The first effective treatment for AL amyloidosis was oral melphalan and prednisone. However, only a quarter of patients achieved a hematologic response (ORR; PR, CR) to this treatment and the median survival was only 12-18 months [23]. High-dose melphalan followed by autologous stem cell transplantation (HDM/SCT) was explored in AL amyloidosis based on its success in treating multiple myeloma. A pilot study of five patients conducted at Boston University was published in 1996 and demonstrated the safety and efficacy of this approach [24]. The phase II study (N = 25) was subsequently reported and demonstrated a complete hematologic response in 62% (13/21) of evaluable patients and organ improvement in 65% of patients [25].

Although HDM/SCT effectively reduces clonal disease and circulating light chains in patients with AL amyloidosis, the toxicity of this approach must be appreciated. The average treatment related mortality (TRM) in four single center studies is 21% but has been reported as high as 39%[26]. Patients with cardiac involvement and autonomic dysfunction are particularly susceptible to fluid shifts and hypotension as the result of high-dose G-CSF and must be monitored during all phases of treatment including mobilization/collection. Patients with cardiac amyloid can experience critical arrhythmias or sudden death during stem cell infusion presumably related to the toxicity of the DMSO preservative. Washing the cells prior to infusion may reduce this risk and is a common practice at some centers. Cardiac staging has helped to minimize TRM by identifying patients susceptible to complications of HDM/SCT [27,28]. With careful patient selection and vigilant attention to supportive strategies, HDM/SCT can be safe but should only be performed at experienced centers.

Two large studies from experienced centers confirmed the utility of HDM/SCT as a treatment for AL amyloidosis. At Boston University, 312 patients with AL amyloidosis were treated with HDM/SCT at 200 mg/m^2 ^or 140 mg/m^2 ^based on age and cardiac status. Utilizing a multidisciplinary team for peri-transplant management, TRM was reduced to 14% in these selected patients [16]. In this series, the median survival for those who achieved CR was more than 10 years compared to 50 months for those who did not achieve CR [29]. A second large patient series from the Mayo Clinic reported 434 patients with AL amyloidosis treated with HDM/SCT over 14 years [6]. A hematologic response was seen in 76% of patients including 39% who achieved CR. Treatment related mortality was 10%[6]. As seen in the Boston series, the strongest predictor of outcome was hematologic CR. The median survival was not reached for those who achieved CR, compared to 107 months for those with PR and 32 months for those with no response (p < 0.001)[6].

At Memorial Sloan-Kettering Cancer Center, risk-adapted chemotherapy dosing has been combined with post transplant consolidation in an effort to minimize toxicity and maintain efficacy [30]. In a series of trials, patients with newly diagnosed AL amyloidosis were treated with melphalan 100, 140 or 200 mg/m^2 ^based on age, renal function and cardiac involvement. Patients with persistent clonal disease following SCT received consolidation, either thalidomide and dexamethasone or bortezomib and dexamethasone, in sequential studies. The low TRM (4% and 11% respectively) in both trials suggests that this approach is safe. With thalidomide-based consolidation, the overall response rate at 12 months was 71% (including 36% CR)[31]; and was 96% (65% CR) when bortezomib-based therapy was used [32]. Organ improvement one year post transplant was noted in 44% and 60% of patients with thalidomide and bortezomib-based consolidation, respectively [31,33]. Notably, organ function continued to improve over time and increased from 60% to 88% at 24 months after SCT in the latter study [34].

Despite the reported efficacy, the use of HDM/SCT in AL amyloidosis remains controversial. A pivotal trial highlighting this controversy was reported by the Intergroupe Francophone du Myeloma (IFM) in 2007 [35]. Among 29 centers, 100 patients were randomized to either conventional chemotherapy using oral melphalan and dexamethasone or HDM/SCT. Although hematologic response rates were similar (68% vs. 67%) in both treatment groups; at three years of follow up, the median OS was superior in the conventional chemotherapy arm (57 vs. 22 months; *P *= 0.04)[35]. Treatment related mortality was unusually high (24%) in the first 100 days following HDM/SCT suggesting that patients may not have been suitable candidates for transplant. In addition, the study was performed at multiple centers, some with very limited experience in caring for patients with amyloidosis. A landmark analysis examining only patients alive six months post treatment showed no difference in the overall survival between the two groups (*P *= 0.38) leading the authors to conclude that there is no benefit of HDM/SCT over conventional chemotherapy. However, with almost 20% of patients excluded from this analysis (9 in the SCT arm) in addition to 13 patients who never received the prescribed HDM (10 died, and 3 others excluded), the number of evaluable patients who underwent SCT is small and insufficient to suggest HDM/SCT should be abandoned.

### Non-transplant approaches

While HDM/SCT is an effective way of achieving rapid, hematologic responses, only 20-25% of patients presenting with AL amyloidosis are eligible for such aggressive treatment [6]. Strategies for those not eligible for transplantation have largely been alkylator-based oral regimens but have now may include novel agents such as immunomodulatory drugs or proteasome inhibitors.

Melphalan and prednisone became the standard of care when superior outcomes were demonstrated as compared to colchicine [23]. Although objective responses could be demonstrated, these were often delayed (median time to response close to 1 year), and only seen in the minority of patients (< 30%). Because responses are slow, organ progression may occur during the initial months of therapy. In patients who remain clinically stable, it is often difficult to know if a patient is destined to fail alkylator based therapy or whether it is too early to abandon the approach. Despite these limitations, alkylating agents can be useful in patients ineligible for aggressive therapy. Even patients with severe cardiac involvement may benefit from continuous, daily, oral melphalan as a palliative measure [36].

Although high-dose dexamethasone regimens accelerate response times in patients with AL amyloidosis, the usual schedule of dexamathasone (40 mg on days 1-4, 9-12, 17-20) is toxic for these patients [37]. A modified schedule of dexamethasone (40 mg days 1-4) was developed and response rates are promising when used in combination with melphalan. In 46 patients treated with oral melphalan and high dose dexamethasone (MDex), 31 (67%) achieved a hematologic response and 15 (33%) achieved a complete response. Twenty-two (48%) patients experienced improvement in organ function with a median time to response of 4.5 months. The day 100 mortality was only 4% and adverse effects were seen in 11% of patients [38]. An update of this study showed the median progression free and overall survival was 3.8 and 5.1 years, respectively [39]. Similar to high dose chemotherapy, the survival was longer for patients who responded to therapy (median not reached) compared to those who did not respond (2.1 years)[39]. Subsequent studies confirmed the activity of this regimen, although outcomes for patients with advanced cardiac disease remain poor with a median overall survival of 10.5 months [40]. Current studies seek to improve the efficacy of oral melphalan and dexamethasone by adding a third agent (such as thalidomide, lenalidomide or bortezomib) to this combination [41-43]. The combination of bortezomib, melphalan and dexamethasone (BMDex) is being compared in a randomized fashion to standard MDex as upfront treatment for patients with AL amyloidosis who are ineligible or refuse SCT. Two distinct alkylating agents, cyclophosphamide and bendamustine, in combination with corticosteroids and novel agents are also being investigated.

### Novel agents

Thalidomide was the first novel agent explored for AL amyloidosis due to its efficacy in multiple myeloma. A phase I/II dose escalation trial using thalidomide in patients previously treated with melphalan and dexamethasone found the agent to have activity but with significant toxicity and the starting dose in AL amyloidosis should be no higher than 50 mg [44]. Lenalidomide, a second generation immunomodulatory (IMID) agent, has been combined with dexamethasone for the treatment of AL amyloidosis. Hematologic response rates were 67% in a phase II trial and were associated with organ responses [45]. The median time to response was 6 cycles (range, 3 to 6). A reduced dose of 15 mg/day was better tolerated than the daily dose of 25 mg/day used in multiple myeloma. Side effects include cytopenias, rash, fatigue, muscle cramping and venous thrombosis. Patients require anti-thrombotic prophylaxis similar to patients with multiple myeloma [46]. Phase I/II studies combining lenalidomide and dexamethasone with either melphalan or cyclophosphamide are ongoing but myelosuppression may be limiting [41]. Pomalidomide, the newest IMID being investigated clinically, was associated with a 47% response rate in extensively pre-treated patients with AL amyloidosis [47]. Severe adverse events ≥ grade 3 were seen in 56% of patients with neutropenia being most common. Increases in BNP/NT-proBNP with Imid-based regimens were initially concerning for cardiac decompensation and led to early discontinuation of therapy. It remains unclear whether this elevation represents true cardiac toxicity, fluid retention or is entirely clinically insignificant. However, it makes assessing organ response very challenging.

Targeting the proteasome, the cellular machinery largely responsible for protein homeostasis was rational based on the misfolded nature of proteins in AL amyloidosis. Bortezomib, a reversible inhibitor of the 26S proteasome has been studied in a phase I/II dose escalation trial as a single agent. Doses up to 1.6 mg/m^2 ^weekly and 1.3 mg/m^2 ^on a biweekly schedule were well tolerated in patients with relapsed disease [48]. Seventy patients were treated on the phase II portion, the majority on the biweekly schedule with responses seen in 67% of patients [49] illustrating the single agent activity of bortezomib in AL amyloidosis. The time to first response was rapid (1.2 months) with a median time to CR of 2.3 months [48]. Overall treatment was safe with peripheral neuropathy seen in 45% of patients [48]. When 1.3 mg/m^2 ^biweekly was used in combination with dexamethasone, patients with relapsed disease (N = 7) or those ineligible for HDM/SCT (N = 11) had a 94% response rate including a 44% CR. Organ improvement occurred in 28% of patients. Again, hematologic responses were rapid (< 1 month) as was time to organ improvement (4 months). Neurotoxicity occurred in 67% of patients but was > grade 2 in only 7%. Dexamethasone toxicity was transient and manageable and the main reason for discontinuation of treatment was adverse effects from bortezomib in 44% of patients [50].

Bortezomib has been combined with oral melphalan and dexamethasone (BMDex) to treat AL amyloidosis with promising response frequency (83%) in untreated (N = 13) and relapsed (N = 17) patients [42,43]. The randomized trials comparing BMDex to standard MDex are currently enrolling in the United States and in Europe and have the potential to change the standard of care for newly diagnosed AL amyloid patients. Cyclophosphamide, bortezomib and dexamethasone (CyBor-D) also demonstrates significant activity in AL amyloidosis with hematologic responses in 93% of untreated (N = 8) and relapsed (N = 7)patients [51]. Second and third generation proteasome inhibitors are in earlier stages of development including carfilzomib, an irreversible proteasome inhibitor with known activity in multiple myeloma and the orally bioavailable agent MLN9708.

### Immunotherapy

The notion that amyloid deposits persist due to their recognition as "self'' by the immune system, protected from effective immune attack, has led to strategies that harness the immune system to target amyloid deposits directly, the precursor amyloid-forming protein or alternately the pathologic plasma cell. Amyloid fibrils, regardless of etiology, share constituent non-fibrillary proteins including serum amyloid P (SAP), a calcium-dependent glycoprotein universally concentrated in amyloid deposits [13]. Because SAP stabilizes amyloid fibrils and promotes fibrillogenesis, SAP was considered a potential therapeutic target and several strategies have emerged. A novel compound, CPHPC ((R) -1-[6-[(R)-2-Carboxy-pyrrolidin-1yl]-6-oxo-hexanoyl] pyrrolidine-2 carboxylic acid) is directed at SAP specifically. CPHPC binds to circulating SAP to form complexes that are rapidly cleared by the liver [52]. In 31 patients with systemic amyloidosis, subcutaneous CPHPC resulted in significant decreases in the circulating SAP concentration; however, tissue-bound SAP remained present in amyloid deposits in tissues [53,54]. To target residual bound SAP, anti SAP immunoglobulin-G (IgG) antibodies have been generated [55]. In a murine system, transgenic mice with human SAP and amyloid deposition in the liver and spleen were treated first with CPHPC to eliminate circulating human SAP followed by a single dose of the anti-SAP antibody. By 24 hours following anti-SAP IgG injection, visceral amyloid deposits were densely infiltrated by inflammatory cells and by 7 days almost all amyloid in the liver and spleen was destroyed. Amyloid clearance was largely complete by day 16 following treatment and the normal architecture of liver and spleen were restored [55]. Based on these studies, CPHPC in combination with a fully humanized monoclonal anti-human SAP is currently being studied in early phase clinical trials in Europe and may be applicable to all forms of amyloid.

Targeting the kappa and lambda light chain has also been explored, and investigators at the University of Tennessee have generated monoclonal antibodies by immunizing mice with human light chain fibrils [56]. Interestingly, these antibodies recognize an epitope common to the beta pleated sheet structure of AL and other amyloid proteins and may also have broader therapeutic implications. Using an in-vivo animal model in which human amyloidomas were produced in mice [56], and radioactively labeled monoclonal antibodies localized only to the tumor [57]. Subsequent studies showed that the amyloidomas could rapidly be eliminated following antibody administration [56]. One prototypic antibody, IgG1k mAb 11-1F4, has been chimerized and is being studied in a phase I/II study.

Immunotherapeutic approaches directed at the pathologic plasma cell are also being investigated. Studies exploring the expression of cancer testis antigen (CTA) on the plasma cells of patients with multiple myeloma have stimulated similar research in AL amyloidosis. CTAs are a class of proteins found on a variety of tumor cells but are otherwise restricted to testicular germ cells and the placenta. The pathologic plasma cells of multiple myeloma commonly express two specific CT antigens, CT7 and MAGEA3 as demonstrated by immunohistochemistry and RT-PCR and expression is increased with advanced disease and higher degree of plasma cell proliferation [58]. An antigen-specific cancer immunotherapeutic combining recombinant MAGEA3 and an adjuvant has been developed and is in phase I testing as post transplant consolidation in patients with multiple myeloma. In AL amyloidosis, CT-7 expression has been confirmed by immunohistochemistry in 60% of patients studied [59]. CT-7 DNA and dendritic cell (DC) vaccines are currently being developed and may have promise for AL amyloid patients in the future.

The graft versus tumor effect that follows allogeneic SCT is the most potent form of immunotherapy. Due to amyloid related organ disease, toxicity of allogeneic SCT can be substantial in AL patients [60]. However, the success of this strategy in small numbers of patients provides proof of principal that antitumor immune effects may be important in AL patients [61-64]. A review of 19 patients from the European Group for Blood and Marrow Transplantation (EBMT) registry reported one year OS and progression free survival (PFS) as 60% and 53% respectively; at two years OS and PFS were 52% and 46%, respectively [60]. A high TRM of 40% was seen among all patients. Favorable performance status, non-total body irradiation (TBI) - based conditioning and the use of a reduced intensity regimen were associated with improved outcomes. Patients treated with ex-vivo T-cell depletion had worse outcomes compared to those treated with conventional grafts and chronic graft versus host disease (GVHD) was observed in the 5 of 7 evaluable patients who achieved a CR suggesting an immunologic graft versus disease effect [60]. As with other treatments for AL amyloidosis, CR was the main predictor of long term survival. Transplant physicians are now charged with developing a well tolerated conditioning regimen to be combined with T-cell manipulation, perhaps with the early introduction of donor lymphocyte infusion in order to spare toxicity and take advantage of a graft versus tumor affect in patients with relapsed disease.

## Conclusion

AL amyloidosis is a rare and potentially devastating disease that is likely under diagnosed. Advances in diagnostic techniques and the use of cardiac biomarkers for staging and free light chains to grade response have improved care. For newly diagnosed patients with stage I and II disease, aggressive treatment with HDM/SCT is warranted because the approach is effective and results in rapid hematologic responses; however, the toxicity of this approach must be appreciated and a risk adapted dosing should be adopted. Treatment for transplant ineligible patients including those with stage III or other advanced organ disease involvement is evolving and may include the use of oral alkylating agents, corticosteroids as well as novel agents in different combinations. The proteasome inhibitor bortezomib has single agent activity in Al amyloidosis and when combined with chemotherapy or administered following SCT, has resulted in the highest response rates to date. Second and third generation proteasome inhibitors are being investigated. For relapsed and refractory patients, newer agents and novel approaches using immunotherapy are being explored.

Referral to a center of excellence experienced in caring for patients with amyloid related organ dysfunction is essential because patients often require the expertise of a multidisciplinary team. Although hematologic responses have become more frequent, organ improvement evolves over months to years so management of patients requires vigilant attention to supportive therapies. In addition, access to investigational approaches is likely to be available only at these centers. While the explosion of novel agents with activity in multiple myeloma holds promise for the care of patients with AL amyloidosis, a commitment specifically to the clinical investigation of treatment for AL amyloidosis is critical in order to improve patient outcomes.

## Competing interests

The authors declare that they have no competing interests.

## Authors' contributions

The present manuscript was drafted by MR and revised by HL. All authors read and approved the final manuscript.
